# Impact of clinical outcomes and imaging measures on health-related quality of life in secondary progressive MS

**DOI:** 10.1177/13524585211063623

**Published:** 2021-12-30

**Authors:** Marcus W Koch, Jop Mostert, Pavle Repovic, James D Bowen, Eva Strijbis, Bernard Uitdehaag, Gary Cutter

**Affiliations:** Department of Clinical Neurosciences, University of Calgary, Calgary, AB, Canada/Department of Community Health Sciences, University of Calgary, Calgary, AB, Canada; Department of Neurology, Rijnstate Hospital, Arnhem, The Netherlands; Multiple Sclerosis Center, Swedish Neuroscience Institute, Seattle, WA, USA; Multiple Sclerosis Center, Swedish Neuroscience Institute, Seattle, WA, USA; Department of Neurology, MS Center Amsterdam, Amsterdam University Medical Centers, Amsterdam, The Netherlands; Department of Neurology, MS Center Amsterdam, Amsterdam University Medical Centers, Amsterdam, The Netherlands; Department of Biostatistics, The University of Alabama at Birmingham, Birmingham, AL, USA

**Keywords:** Secondary progressive MS, health-related quality of life, outcome measures, MRI, disability

## Abstract

**Background:**

Health-related quality of life (HRQOL) outcomes are often included as secondary outcomes in clinical trials in secondary progressive MS (SPMS), but little is known about the longitudinal association of HRQOL and clinical and imaging outcome measures in SPMS.

**Objective:**

To assess the association of change in clinical and imaging outcomes with HRQOL in people with SPMS.

**Methods:**

We used data from ASCEND, a large randomized controlled trial (*n* = 889), to investigate the association of significant worsening on the Expanded Disability Status Scale (EDSS), Timed 25 Foot Walk (T25FW), Nine Hole Peg Test (NHPT), Symbol Digit Modalities Test (SDMT), and change in lesional and volumetric imaging outcomes with significant worsening on the 36-Item Short Form Health Survey (SF-36) and the Multiple Sclerosis Impact Scale (MSIS-29) during 2 years of follow-up using logistic regression models.

**Results:**

HRQOL measures were most associated with EDSS and T25FW, less so with NHPT and SDMT, and not associated with lesional and volumetric imaging outcomes.

**Discussion:**

Worsening of the EDSS and T25FW was associated with two commonly used HRQOL measures. These outcomes therefore appear to be more patient relevant than either the NHPT or SDMT in the context of a 2-year clinical trial.

## Introduction

Health-related quality of life (HRQOL) in multiple sclerosis (MS) depends on a variety of factors including physical disability, cognitive function, social support, hopefulness, and resilience.^1^ While HRQOL is by its nature difficult to measure in a summary score, its inclusion as an outcome measure in clinical trials adds the dimension of subjective patient experience and patient-relevance to the purely objective measurement of disability and magnetic resonance imaging (MRI) changes. Clinical trials in secondary progressive MS (SPMS) therefore often include HRQOL measures as secondary outcomes.

HRQOL can be measured with a number of standardized questionnaires. Two of the most widely used tools are the Medical Outcomes Study Short Form Health Survey (SF-36),^2^ a 36-item questionnaire used since the 1990s, and the more recently developed Multiple Sclerosis Impact Scale (MSIS-29),^3^ a 29-item questionnaire with MS-specific items. Both these tools offer an evaluation of physical and psychological HRQOL through separate summary scores for psychological and physical HRQOL.

Observational studies on HRQOL in MS have been conducted since the 1990s and show that HRQOL is generally worse in people with MS compared to the general population,^4^ worse in MS than in comparable chronic inflammatory conditions,^5^ and worse in individuals with a progressive disease course compared to those with relapsing-remitting MS.^6^ The literature on the relationship of disability and HRQOL is dominated by smaller cross-sectional studies. Such studies showed that disability measured with the Expanded Disability Status Scale (EDSS),^7^ the most commonly used primary outcome measure in clinical trials in MS, as well as with the newer measures Timed 25 Foot Walk (T25FW),^8^ Nine Hole Peg Test (NHPT),^9^ and the cognitive outcome measure Symbol Digit Modalities Test (SDMT),^10^ correlates moderately with HRQOL.^6,11–14^

While these cross-sectional studies are informative, there is a lack of large longitudinal studies investigating the impact of significant change in these physical and cognitive outcomes and significant change in HRQOL. Similarly, it is also unclear whether changes in imaging outcome measures such as whole brain or gray matter atrophy are related to change in HRQOL.

In this investigation, we use a large dataset of a recent phase-3 randomized controlled trial in SPMS to investigate the association of change in clinical and imaging outcomes with change of SF-36 and MSIS-29 scores over 2 years of follow-up.

## Methods

### ASCEND dataset

The ASCEND dataset is described in detail in the original publication of the trial.^15^ ASCEND is a randomized, double blind, placebo-controlled, two-arm trial of natalizumab treatment in SPMS. The inclusion criteria were age 18–58 years inclusive, SPMS for 2 or more years, disability worsening in the year before inclusion, a screening EDSS score of 3.0–6.5 inclusive, and a Multiple Sclerosis Severity Score^16^ of 4 or more. It excluded patients with a clinical relapse in the 3 months before inclusion. In ASCEND, SPMS was defined as a relapsing-remitting disease followed by the progression of disability independent of or not explained by MS relapses for at least 2 years.

### HRQOL outcomes

Trial participants completed MSIS-29 and SF-36 questionnaires at baseline, and then at 24, 48, 72, and 96 weeks. We calculated the MSIS-29 Physical and Psychological subscores for each time point. The MSIS-29 Psychological and Physical subscores can range from 0 to 100, with higher scores indicating worse HRQOL. We calculated the SF-36 Physical Component Summary (PCS) and Mental Component Summary (MCS) scores for each of these time points. The SF-36 PCS and MCS scores range from 0 to 100, with higher scores indicating better HRQOL. We determined the number of individuals with unconfirmed significant HRQOL worsening at each time point. For the MSIS-29 Physical and Psychological subscores, we defined significant worsening as an increase by 8 or more points compared to baseline.^17^ For the SF-36 PCS and MCS subscores, we defined a 5 or more point decrease from baseline as significant worsening.^18,19^

### Clinical outcomes

EDSS, T25FW and NHPT were measured at the baseline visit and then every 12 weeks. SDMT was measured at baseline and then every 4 weeks. For this study, we used significant worsening of disability with 3-month confirmation (3 months of confirmed disability progression, 3M CDP) measured at the main study visits every 12 weeks. We determined the percentage of individuals with significant worsening of disability by comparing the baseline and follow-up measurements of EDSS, T25FW, and SDMT. Individuals missing a measurement at baseline, the follow-up time point of interest, or the corresponding 3-month confirmation assessment were excluded from the analysis. We defined significant worsening on the EDSS as an increase of one whole point on the EDSS if the screening EDSS was 5.5 or lower, and of one-half point if the screening EDSS was 6.0 or 6.5 (this definition was used in the original trial). For T25FW and NHPT, we defined significant worsening as a 20% or greater increase from screening. We used a 4-point decrease in the SDMT score as significant worsening, since this margin of worsening is associated with loss of employment in people with MS and generally seen as clinically significant.^20^

### MRI outcomes

Gadolinium-enhanced cranial MRI scans were performed at the screening visit of the trial, and then at 24, 48, 72, and 96 weeks of follow-up. Normalized brain volume (NBV), normalized cortical gray matter volume (NCGMV), and normalized whole gray matter volume (NWGMV) were determined using SIENAX, a segmentation-based cross-sectional method.^21^ The Jacobian integration technique was used to generate percent brain volume change, percent whole GMV change, and percent cortical GMV change on 3-mm-thick slices. The T2 lesion volume and the number and volume of contrast enhancing lesions were assessed for all scans, and the number of new or newly enlarging T2 lesions for all scans after screening. We determined the cumulative number of contrast enhancing lesions (cCEL) and the cumulative number of new or newly enlarging T2 lesions (cNT2) at 24, 48, 72, and 96 weeks.

### Association of HRQOL with disability worsening and change in MRI measures

In the first step, we explored the differences in HRQOL summary scores between participants with and without significant disability worsening at 48 and 96 weeks using Student’s *t*-test. We also explored the differences in HRQOL summary scores between participants with different degrees of MRI change at 48 and 96 weeks using one-way analysis of variance (ANOVA). We categorized the change in volume measures NBV, NCGMV, and NWGMV into five categories: (1) volume increase or no change, (2) up to 0.5% volume loss, (3) between 0.5% and 1% volume loss, (4) between 1% and 1.5% volume loss, and (5) more than 1.5% volume loss. We categorized cNT2 into four categories: (1) None, (2) 1 to 5, (3) 6 to 10, and (4) more than 10. To achieve the greatest sensitivity for discovering associations, we chose not to correct the significance levels for multiple comparisons.

We then used logistic regression models to assess the association of significant HRQOL worsening (dependent variable) and worsening of disability measures and MRI measures of interest (independent predictor variables). Additional independent predictor variables included in the models were as follows: age, sex, treatment arm, the HRQOL summary score at baseline, and the disability measure of interest at baseline or the MRI outcome of interest at screening. We used the R statistical software package for Windows version 4.0.2^22^ for all statistical analyses. Statistical significance was taken to be at the two-tailed 0.05 level.

### Data availability

The data used in this study are available upon request from Biogen. Individual participant data collected during the trial will be shared after anonymization and on approval of a research proposal and data sharing agreement. Research proposals can be submitted online (www.biogenclinicaldatarequest.com).

## Results

### ASCEND dataset

The ASCEND dataset contained data of 889 patients. Table 1 shows their baseline characteristics.

**Table 1.** Baseline clinical and HRQOL characteristics, and imaging characteristics at screening in the ASCEND dataset.

Number of participants	889
Sex (f/m, %)	550 (61.9%) / 339 (38.1%)
Age (median, IQR)	48, 42 to 53
MSIS-29 Physical score (mean, SD)	50.8 (20.2)
MSIS-29 Psychological score (mean, SD)	39.1 (22.4)
SF-36 PCS score (mean, SD)	33.3 (7.9)
SF-36 MCS score (mean, SD)	47.0 (10.6)
EDSS (median, IQR)	6.0, 5.0 to 6.5
T25FW (median, IQR)	11.2, 7.9 to 17.0
NHPT (median, IQR)	30.3, 25.5 to 38.8
SDMT (median, IQR)	39, 30 to 49
Patients with enhancing lesions (n, %)	212, 23.9^a^
NBV [cm^3^] (mean, SD)	1423.9, 83.3
NCGMV [cm^3^] (mean, SD)	513.9, 53.0
NWGMV [cm^3^] (mean, SD)	684.9, 63.8
T2 lesion volume [cm^3^] (mean, SD)	16.9, 17.5

HRQOL: health-related quality of life; IQR: interquartile range; MSIS-29: Multiple Sclerosis Impact Scale; SD: standard deviation; SF-36: 36-Item Short Form Health Survey; PCS: Physical Component Summary; MCS: Mental Component Summary; EDSS: Expanded Disability Status Scale; T25FW: Timed 25 Foot Walk; NHPT: Nine Hole Peg Test; SDMT: Symbol Digit Modalities Test; NBV: normalized brain volume; NCGMV: normalized cortical gray matter volume; NWGMV: normalized whole gray matter volume.

Higher scores on MSIS-29 indicate worse HRQOL, higher scores on the SF-36 indicate better HRQOL.

a *n* = 888.

### HRQOL outcomes

**Figure 1.**
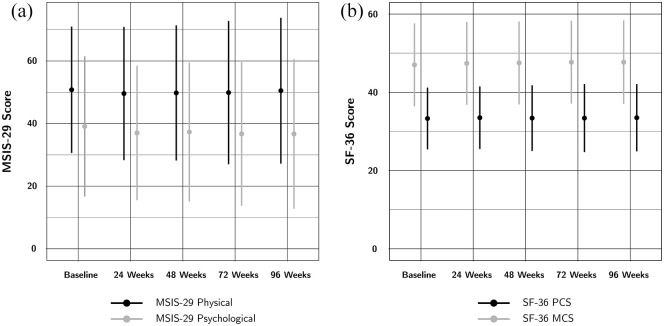
Mean (a) MSIS-29 and (b) SF-36 summary scores at baseline and throughout follow-up. The error bars represent the standard deviation. There is little change in the mean HRQOL subscores throughout the trial.

**Figure 2.**
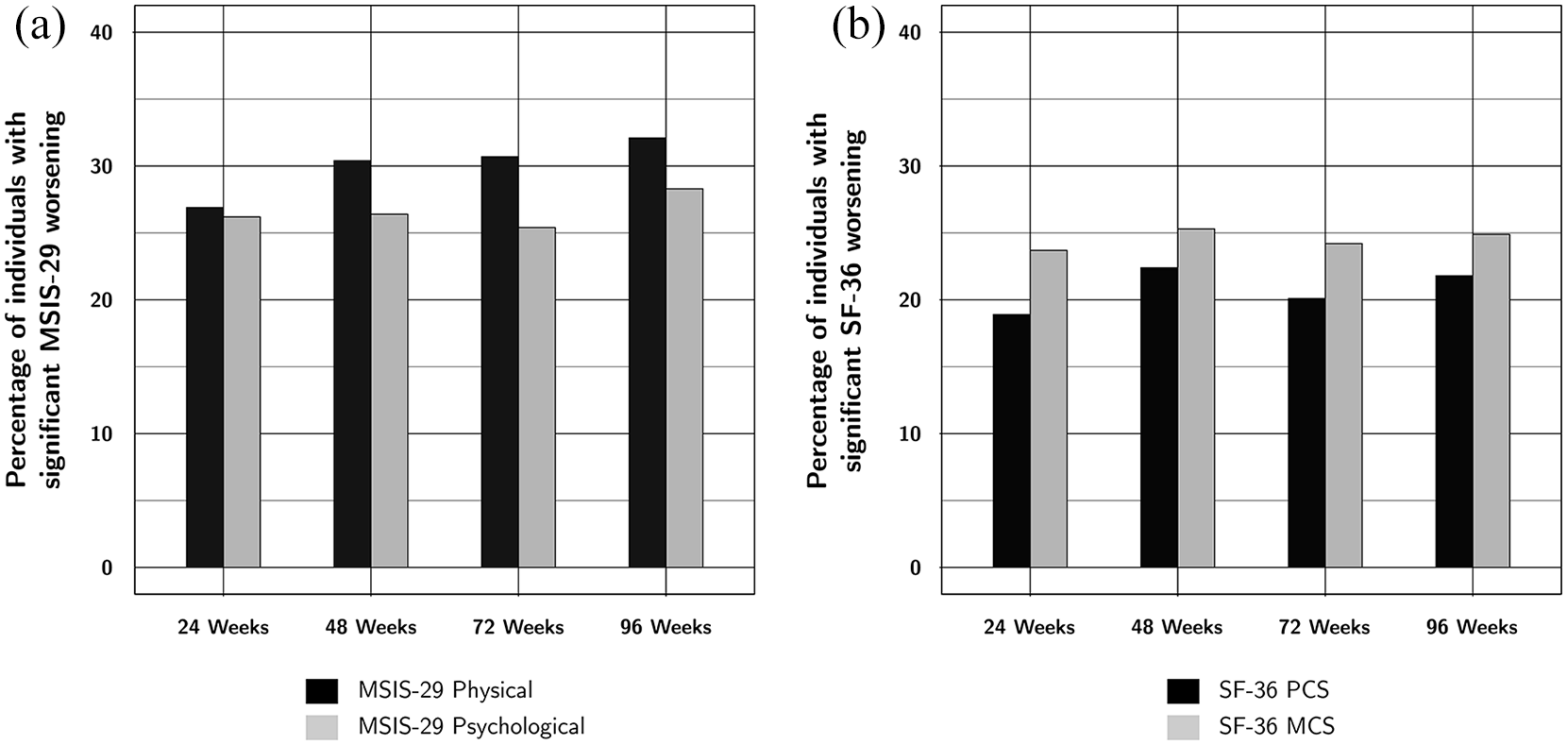
Percentage of trial participants with significant change in (a) MSIS-29 and (b) SF-36 summary scores. The percentage of individuals with significant worsening in the MSIS-29 Physical subscore slightly but steadily increases over the course of the trial. The other subscores show no consistent change throughout follow-up.

Change in the investigated HRQOL outcomes is shown in Table 2 and Figures 1 and 2. The percentage of participants with significant worsening on the MSIS-29 Physical scores increased very slightly and steadily throughout follow-up, from 26.9% at 24 weeks to 32.1% at 96 weeks, whereas the other investigated HRQOL measures showed no consistent change over time (Table 2 and Figure 2). All measures also showed slight increases in the variability of changes.

**Table 2.** Changes in HRQOL, clinical, and MRI measures over 2 years of follow-up.

Outcome	24 weeks	48 weeks	72 weeks	96 weeks
MSIS-29 Physical (mean, SD) Number of observations (*n*)	49.6 (21.3) 786	49.8 (21.6) 733	49.9 (22.9) 684	50.5 (23.3) 648
MSIS-29 Psychological (mean, SD) Number of observations (*n*)	37.0 (21.5) 792	37.3 (22.2) 742	36.7 (23.0) 685	36.7 (23.9) 649
SF-36 PCS (mean, SD) Number of observations (*n*)	33.5 (8.0) 770	33.4 (8.4) 712	33.4 (8.7) 660	33.5 (8.6) 625
SF-36 MCS (mean, SD) Number of observations (*n*)	47.4 (10.6) 770	47.5 (10.6) 712	47.7 (10.6) 660	47.7 (10.7) 625
Percentage of participants with significant worsening (%)				
MSIS-29 Physical	26.9	30.4	30.7	32.1
MSIS-29 Psychological	26.2	26.4	25.4	28.3
SF-36 PCS	18.9	22.4	20.1	21.8
SF-36 MCS	23.7	25.3	24.2	24.9
Percentage of participants with significant worsening (%)				
EDSS 3M CDP	6.8	11.7	14.1	17.7
T25FW 3M CDP	17.9	25.6	25.7	28.6
NHPT 3M CDP	4.1	5.7	6.4	8.2
SDMT 3M CDP	3.4	2.7	3.3	3.2
NBV change (%, SD)	−0.32 (0.5)	−0.53 (0.57)	−0.75 (0.68)	−0.95 (0.76)
NCGMV change (%, SD)	−0.49 (0.72)	−0.74 (0.77)	−0.99 (0.91)	−1.18 (0.96)
NWGMV change (%, SD)	−0.51 (0.65)	−0.73 (0.69)	−0.96 (0.81)	−1.13 (0.86)
T2 lesion volume change (%, SD)	−0.39 (9.07)	−0.13 (13.11)	−0.71 (15.21)	−0.55 (15.01)
cCEL				
Mean, SD	1.34 (5.25)	1.63 (6.65)	1.93 (8.48)	2.21 (10.3)
Median, IQR	0 (0 to 1)	0 (0 to 1)	0 (0 to 1)	0 (0 to 1)
cNT2				
Mean, SD	1.54 (4.32)	2.4 (6.71)	3.18 (8.77)	3.7 (10.04)
Median, IQR	0 (0 to 1)	0 (0 to 2)	0 (0 to 2)	0 (0 to 3)

HRQOL: health-related quality of life; MRI: magnetic resonance imaging; MSIS-29: Multiple Sclerosis Impact Scale; SD: standard deviation; SF-36: 36-Item Short Form Health Survey; PCS: Physical Component Summary; MCS: Mental Component Summary; 3M: 3 months; EDSS: Expanded Disability Status Scale; CDP: unconfirmed disability progression; T25FW: Timed 25 Foot Walk; NHPT: Nine Hole Peg Test; SDMT: Symbol Digit Modalities Test; NBV: normalized brain volume; NCGMV: normalized cortical gray matter volume; NWGMV: normalized whole gray matter volume; cCEL: cumulative number of contrast enhancing lesions; IQR: interquartile range; cNT2: cumulative number of new or newly enlarging T2 lesions.

Higher scores on MSIS-29 indicate worse HRQOL, higher scores on SF-36 indicate better HRQOL.

### Clinical outcomes

Table 2 shows the change in the investigated clinical outcome measures over the 2 years of follow-up. The number of participants with significant worsening on the EDSS, T25FW, and NHPT steadily increased throughout the course of the trial, while there was little change in SDMT. The T25FW had the most worsening events, followed by the EDSS and NHPT.

### MRI outcomes

Table 2 shows the change in the investigated MRI outcomes. NBV, NCGMV, and NWGMV steadily decreased throughout follow-up reaching a mean volume loss of around 1% on all these volume measures at 96 weeks, whereas the T2 lesion volume changed little during follow-up (Table 2). The cCEL and cNT2 steadily increased throughout follow-up (Table 2). All measures also showed slight increases in the variability of changes.

### Association of HRQOL with significant disability worsening and MRI changes

The unadjusted comparisons of HRQOL scores between patients with and without significant disability worsening and by degree of MRI change are shown in Tables 3 and 4. In these unadjusted analyses, worsening of the EDSS and T25FW was most consistently associated with HRQOL, followed by the NHPT. There were no significant differences in HRQOL scores between patients with and without significant worsening on the SDMT.

**Table 3.** Differences in HRQOL scores between patients with and without significant disability worsening, and by category of MRI change at 48 weeks.

		MSIS-29 Physical		MSIS-29 Psychological		SF-36 PCS		SF-36 MCS	
		Mean score (SD)	*p*	Mean score (SD)	*p*	Mean score (SD)	*p*	Mean score (SD)	*p*
EDSS 3M CDP	Yes	55.8 (21.7)	0.005	40.9 (21.8)	0.15	31.6 (7.9)	0.03	44.7 (12.0)	0.02
	No	48.5 (21.3)		36.7 (22.1)		33.8 (8.5)		48.1 (10.1)	
T25FW 3M CDP	Yes	57.2 (20.5)	< 0.0001	39.4 (20.4)	0.19	31.0 (7.7)	< 0.0001	47.2 (11.5)	0.53
	No	46.0 (21.0)		36.6 (22.3)		34.6 (8.5)		47.9 (9.9)	
NHPT 3M CDP	Yes	60.9 (20.2)	0.001	44.6 (22.7)	0.07	30.2 (6.2)	0.003	44.2 (12.7)	0.14
	No	48.0 (21.4)		36.5 (21.7)		34.0 (8.6)		47.9 (10.4)	
SDMT 3M CDP	Yes	41.9 (23.9)	0.93	45.5 (27.5)	0.33	34.5 (6.5)	0.55	45.0 (9.8)	0.29
	No	49.6 (21.5)		36.9 (22.0)		33.5 (8.5)		47.7 (10.5)	
NBV change	⩾ 0%	45.8 (21.9)	0.12	35.1 (23.2)	0.53	33.8 (9.9)	0.91	49.5 (9.8)	0.10
	< 0% to −0.5%	49.5 (21.1)		36.1 (21.6)		33.4 (8.2)		47.7 (10.7)	
	< –0.5% to −1%	51.2 (21.7)		37.9 (21.5)		33.2 (7.8)		47.7 (10.3)	
	< –1% to −1.5%	50.6 (21.8)		41.1 (23.4)		34.3 (8.6)		44.9 (12.2)	
	< –1.5%	55.7 (22.5)		38.7 (21.8)		33. (9.3)		47.1 (12.1)	
NCGMV change	⩾ 0%	47.2 (22.2)	0.26	33.1 (21.7)	0.12	33.0 (8.3)	0.75	48.4 (10.2)	0.32
	< 0% to −0.5%	48.0 (22.7)		37.8 (22.2)		34.2 (9.1)		48.0 (10.5)	
	< –0.5% to −1%	49.7 (20.2)		36.9 (22.0)		33.0 (8.1)		47.5 (11.0)	
	< –1% to −1.5%	52.3 (20.2)		35.9 (20.6)		33.7 (8.5)		48.4 (10.8)	
	< –1.5%	52.1 (23.8)		42.6 (24.0)		33.6 (8.7)		45.6 (10.7)	
NWGMV change	⩾ 0%	47.1 (22.0)	0.13	33.4 (22.0)	0.06	33.1 (8.0)	0.63	48.5 (9.7)	0.02
	< 0% to −0.5%	47.0 (21.5)		35.0 (21.8)		34.1 (8.9)		49.1 (10.4)	
	< –0.5% to −1%	51.1 (21.2)		39.0 (22.0)		32.9 (8.1)		46.6 (11.)	
	< –1% to −1.5%	50.6 (20.9)		35.3 (20.6)		34.0 (9.1)		48.9 (10.2)	
	< –1.5%	53.7 (23.2)		42.9 (24.2)		33.5 (8.1)		44.8 (11.4)	
T2 lesion volume change	⩽ 0%	49.2 (20.9)	0.11	36.7 (24.7)	0.82	33.5 (8.4)	0.08	47.6 (9.8)	0.99
	> 0% to 2.5%	46.6 (22.8)		38.4 (24.7)		34.8 (8.4)		47.3 (12.2)	
	> 2.5% to 5%	52.5 (22.3)		38.2 (24.2)		33.2 (9.4)		47.9 (11.5)	
	> 5%	52.6 (21.7)		38.7 (22.4)		32.0 (7.9)		47.5 (10.7)	
cCEL	None	49.8 (22.0)	0.10	37.9 (22.9)	0.47	32.9 (8.4)	0.09	47.7 (10.5)	0.42
	1 to 5	48.4 (20.8)		35.6 (21.1)		34.6 (8.4)		48.1 (10.6)	
	6 to 10	56.3 (17.2)		42.4 (22.0)		31.9 (7.9)		45.4 (10.9)	
	More than 10	57.2 (23.6)		35.7 (19.6)		32.4 (9.0)		45.2 (10.0)	
cNT2	None	50.0 (21.4)	0.27	38.2 (22.7)	0.22	32.7 (8.3)	0.02	47.9 (10.4)	0.78
	1 to 5	48.5 (21.7)		36.2 (21.9)		34.8 (8.8)		47.0 (10.6)	
	6 to 10	52.1 (21.1)		30.7 (19.5)		34.5 (8.9)		48.5 (11.0)	
	More than 10	55.3 (21.6)		40.7 (20.0)		31.9 (6.6)		47.3 (10.9)	

HRQOL: health-related quality of life; MRI: magnetic resonance imaging; MSIS-29: Multiple Sclerosis Impact Scale; SF-36: 36-Item Short Form Health Survey; PCS: Physical Component Summary; MCS: Mental Component Summary; SD: standard deviation; EDSS: Expanded Disability Status Scale; 3M: 3 months; CDP: confirmed disability progression; T25FW: Timed 25 Foot Walk; NHPT: Nine Hole Peg Test; SDMT: Symbol Digit Modalities Test; NBV: normalized brain volume; NCGMV: normalized cortical gray matter volume; NWGMV: normalized whole gray matter volume; cCEL: cumulative number of contrast enhancing lesions; cNT2: cumulative number of new or newly enlarging T2 lesions.

Higher scores on MSIS-29 indicate worse HRQOL, higher scores on SF-36 indicate better HRQOL.

**Table 4.** Differences in HRQOL scores between patients with and without significant disability worsening, and by category of MRI change at 96 weeks.

		MSIS-29 Physical		MSIS-29 Psychological		SF-36 PCS		SF-36 MCS	
		Mean score (SD)	*p*	Mean score (SD)	*p*	Mean score (SD)	*p*	Mean score (SD)	*p*
EDSS 3M CDP	Yes	59.0 (22.2)	< 0.0001	43.5 (24.3)	0.002	30.5 (7.8)	< 0.0001	45.6 (11.5)	0.02
	No	48.3 (22.9)		35.2 (23.6)		34.3 (8.6)		48.6 (10.4)	
T25FW 3M CDP	Yes	56.2 (21.5)	< 0.0001	41.6 (24.0)	0.003	31.7 (7.8)	< 0.0001	46.9 (10.7)	0.14
	No	46.0 (22.6)		34.5 (23.1)		35.0 (8.7)		48.3 (10.4)	
NHPT 3M CDP	Yes	62.1 (25.1)	0.0005	41.0 (29.0)	0.33	32.3 (8.0)	0.16	48.2 (12.1)	0.89
	No	48.2 (22.5)		36.3 (23.3)		34.0 (8.6)		47.9 (10.5)	
SDMT 3M CDP	Yes	59.3 (22.5)	0.08	50.3 (27.0)	0.05	32.4 (8.2)	0.49	44.4 (13.1)	0.24
	No	49.7 (23.3)		35.9 (23.9)		33.7 (8.6)		48.2 (10.6)	
NBV change	⩾ 0%	49.1 (24.9)	0.11	36.9 (27.3)	0.40	32.8 (8.8)	0.68	48.9 (9.3)	0.06
	< 0% to −0.5%	47.8 (20.9)		33.5 (22.6)		33.3 (8.6)		49.1 (11.2)	
	< –0.5% to −1%	48.5 (23.0)		37.3 (23.0)		34.4 (9.0)		47.9 (10.1)	
	< –1% to −1.5%	50.3 (24.5)		39.2 (23.7)		34.1 (8.7)		47.3 (10.2)	
	< –1.5%	55.5 (22.6)		40.0 (25.3)		33.3 (8.1)		44.9 (12.3)	
NCGMV change	⩾ 0%	49.3 (25.3)	0.08	37.0 (25.9)	0.29	32.9 (8.6)	0.69	48.4 (10.5)	0.11
	< 0% to −0.5%	45.4 (20.5)		33.8 (23.2)		34.7 (7.9)		49.0 (10.2)	
	< –0.5% to −1%	47.4 (22.5)		34.6 (23.1)		34.5 (9.3)		49.3 (10.8)	
	< –1% to −1.5%	51.1 (22.6)		40.4 (23.4)		33.6 (8.5)		46.6 (10.6)	
	< –1.5%	53.4 (24.1)		38.7 (24.4)		33.4 (8.6)		46.3 (11.1)	
NWGMV change	⩾ 0%	49.7 (24.9)	0.01	36.9 (26.1)	0.27	32.5 (8.4)	0.39	48.6 (10.5)	0.09
	< 0% to −0.5%	44.3 (20.6)		33.4 (23.9)		34.8 (7.9)		49.5 (10.2)	
	< –0.5% to −1%	48.1 (22.7)		35.3 (22.7)		34.8 (9.4)		48.2 (10.6)	
	< –1% to −1.5%	49.9 (23.0)		38.2 (22.8)		33.2 (8.6)		47.7 (10.8)	
	< –1.5%	55.2 (23.6)		40.6 (25.0)		33.5 (8.5)		45.6 (11.1)	
T2 lesion volume change	⩽ 0%	50.5 (23.2)	0.60	36.9 (22.9)	0.93	33.7 (8.4)	0.07	47.6 (10.4)	0.54
	> 0% to 2.5%	48.6 (22.7)		38.3 (25.2)		35.3 (8.7)		46.4 (10.0)	
	> 2.5% to 5%	47.8 (24.3)		36.7 (23.3)		33.8 (8.6)		48.3 (11.9)	
	> 5%	52.1 (23.5)		35.9 (26.3)		32.2 (8.7)		48.5 (11.1)	
cCEL	None	50.1 (23.2)	0.05	36.5 (23.7)	0.04	33.4 (8.5)	0.13	48.0 (10.8)	0.04
	1 to 5	48.4 (23.1)		36.1 (24.4)		34.5 (9.0)		47.8 (10.3)	
						30.7 (7.3)			
	6 to 10	61.3 (21.3)		50.9 (21.7)				42.3 (8.4)	
	More than 10	51.1 (25.9)		36.0 (25.3)		34.0 (7.5)		49.7 (12.7)	
cNT2	None	50.3 (22.7)	0.97	37.5 (23.2)	0.58	33.1 (8.2)	0.12	47.9 (10.5)	0.94
	1 to 5	50.5 (24.7)		34.6 (24.4)		34.0 (9.4)		47.6 (10.9)	
	6 to 10	49.2 (24.2)		36.9 (29.5)		36.4 (9.8)		47.0 (11.1)	
	More than 10	51.5 (23.5)		39.6 (23.6)		33.2 (7.8)		47.3 (11.2)	

HRQOL: health-related quality of life; MRI: magnetic resonance imaging; MSIS-29: Multiple Sclerosis Impact Scale; SF-36: 36-Item Short Form Health Survey; PCS: Physical Component Summary; MCS: Mental Component Summary; SD: standard deviation; EDSS: Expanded Disability Status Scale; 3M: 3 months; CDP: confirmed disability progression; T25FW: Timed 25 Foot Walk; NHPT: Nine Hole Peg Test; SDMT: Symbol Digit Modalities Test; NBV: normalized brain volume; NCGMV: normalized cortical gray matter volume; NWGMV: normalized whole gray matter volume; cCEL: cumulative number of contrast enhancing lesions; cNT2: cumulative number of new or newly enlarging T2 lesions.

Higher scores on MSIS-29 indicate worse HRQOL, higher scores on SF-36 indicate better HRQOL.

At 48 weeks, individuals with significant worsening on the EDSS, T25FW, and NHPT also had significantly worse HRQOL as measured with the MSIS-29 Physical and SF-36 PCS. Worsening on the EDSS was also associated with worse SF-36 MCS scores. cNT2 at 48 weeks was also associated with worse SF-36 PCS, but this association was inconsistent, with individuals with more than 10 cNT2 lesions achieving better HRQOL than those with fewer cNT2 (Table 3).

At 96 weeks, individuals with significant worsening on the EDSS had significantly worse HRQOL as measured on all subscores. Worsening on the T25FW was associated with worse HRQOL as measured on the MSIS-29 Physical, MSIS-29 Psychological, and SF-36 PCS. Worsening on the NHPT was associated with worse MSIS-29 Physical HRQOL alone. Neither worsening on the SDMT nor any of the MRI outcomes were associated with the significant differences in HRQOL (Table 4).

After adjustment for other co-variables in the logistic regression models, we found that the EDSS and T25FW worsening were consistently associated with worsening HRQOL as measured with both the MSIS-29 and SF-36 at 48 and 96 weeks. NHPT worsening was less consistently associated with MSIS-29 and SF-36 worsening, and SDMT worsening was inconsistently associated with MSIS-29, but not SF-36 worsening. None of the investigated MRI outcomes were associated with HRQOL worsening in this trial. In most of these models, the HRQOL measure at baseline was significantly associated with HRQOL worsening, in the sense that better HRQOL at baseline was associated with a higher risk of HRQOL worsening at follow-up. This finding most likely represents regression toward the mean (Table 5).

**Table 5.** Results of the logistic regression models investigating the association of change in clinical and imaging outcomes with significant worsening on the MSIS-29 and SF-36 subscales.

Predictor variable	MSIS-29 Physical worse		MSIS-29 Psychological worse		SF-36 PCS worse		SF-36 MCS worse	
	48 weeks	96 weeks	48 weeks	96 weeks	48 weeks	96 weeks	48 weeks	96 weeks
EDSS 3M CDP at 48 weeks	**3.03** (1.78 to 5.18)	**2.98** (1.72 to 5.18)	1.61 (0.89 to 2.85)	**2.55** (1.46 to 4.47)	**3.09** (1.73 to 5.48)	**2.78** (1.49 to 5.10)	**1.99** (1.33 to 3.43)	1.26 (0.68 to 2.28)
EDSS 3M CDP at 96 weeks	**3.26** (2.03 to 5.25)	**3.70** (2.34 to 5.89)	**1.83** (1.13 to 2.96)	**2.29** (1.43 to 3.64)	**3.07** (1.81 to 5.22)	**2.87** (1.71 to 4.80)	**1.79** (1.09 to 2.93)	1.56 (0.94 to 2.54)
T25FW 3M CDP at 48 weeks	**3.55** (2.36 to 5.36)	**3.14** (2.07 to 4.81)	**1.96** (1.23 to 3.09)	1.22 (0.77 to 1.93)	**2.70** (1.71 to 4.29)	**2.06** (1.26 to 3.35)	**2.24** (1.46 to 3.42)	**1.72** (1.09 to 2.72)
T25FW 3M CDP at 96 weeks	**2.45** (1.61 to 3.76)	**2.50** (1.66 to 3.77)	**2.05** (1.32 to 3.19)	**2.10** (1.37 to 3.24)	**2.24** (1.36 to 3.69)	**1.64** (1.00 to 2.64)	**1.79** (1.13 to 2.83)	1.38 (0.87 to 2.17)
NHPT 3M CDP at 48 weeks	**2.99** (1.38 to 6.55)	**2.56** (1.16 to 5.66)	**3.00** (1.30 to 6.84)	**2.32** (1.02 to 5.16)	**3.17** (1.33 to 7.32)	2.12 (0.85 to 4.98)	**2.78** (1.20 to 6.22)	1.92 (0.79 to 4.37)
NHPT 3M CDP at 96 weeks	1.01 (0.96 to 3.70)	1.58 (0.79 to 3.05)	1.26 (0.57 to 2.62)	1.35 (0.64 to 2.71)	1.73 (0.75 to 3.73)	1.34 (0.61 to 2.76)	1.36 (0.61 to 2.81)	1.08 (0.51 to 2.15)
SDMT 3M CDP at 48 weeks	1.47 (0.52 to 3.97)	**3.30** (1.10 to 10.49)	**6.91** (2.00 to 27.64)	**5.44** (1.59 to 21.39)	0.73 (0.16 to 2.44)	0.67 (0.10 to 2.66)	0.75 (0.17 to 2.40)	0.57 (0.09 to 2.13)
SDMT 3M CDP at 96 weeks	0.72 (0.21 to 2.07)	**3.24** (1.27 to 8.63)	**3.20** (1.08 to 9.14)	1.85 (0.61 to 5.20)	1.24 (0.27 to 4.05)	0.88 (0.20 to 2.81)	0.94 (0.21 to 3.01)	0.70 (0.16 to 2.20)
NBV change^a^ at 48 weeks	0.95 (0.70 to 1.30)	0.81 (0.58 to 1.12)	0.92 (0.65 to 1.32)	0.91 (0.65 to 1.29)	0.91 (0.63 to 1.32)	1.03 (0.71 to 1.51)	0.76 (0.54 to 1.07)	0.76 (0.53 to 1.08)
NBV change^a^ at 96 weeks	1.11 (0.84 to 1.48)	0.87 (0.67 to 1.15)	0.98 (0.74 to 1.31)	0.97 (0.73 to 1.29)	1.05 (0.76 to 1.48)	1.11 (0.80 to 1.54)	0.77 (0.57 to 1.04)	0.70 (0.52 to 1.05)
NCGMV change^a^ at 48 weeks	0.89 (0.70 to 1.12)	0.85 (0.66 to 1.08)	0.98 (0.76 to 1.30)	0.92 (0.71 to 1.19)	1.09 (0.84 to 1.43)	1.02 (0.77 to 1.36)	0.85 (0.66 to 1.09)	0.79 (0.60 to 1.03)
NCGMV change^a^ at 96 weeks	0.96 (0.78 to 1.18)	0.82 (0.66 to 1.03)	0.90 (0.72 to 1.13)	0.90 (0.72 to 1.12)	1.00 (0.78 to 1.28)	1.03 (0.81 to 1.32)	0.93 (0.74 to 1.16)	0.75 (0.58 to 1.04)
NWGMV change^a^ at 48 weeks	0.83 (0.64 to 1.08)	0.81 (0.61 to 1.06)	1.02 (0.75 to 1.38)	0.89 (0.66 to 1.19)	1.08 (0.80 to 1.46)	1.04 (0.75 to 1.44)	0.82 (0.62 to 1.08)	0.75 (0.55 to 1.01)
NWGMV change^a^ at 96 weeks	0.94 (0.74 to 1.20)	0.76 (0.54 to 1.07)	0.90 (0.70 to 1.16)	0.90 (0.70 to 1.14)	0.98 (0.74 to 1.30)	1.03 (0.79 to 1.36)	0.91 (0.70 to 1.18)	0.76 (0.54 to 1.07)
T2 lesion volume change^a^ at 48 weeks	1.00 (0.99 to 1.02)	1.00 (0.99 to 1.02)	1.01 (0.99 to 1.02)	1.00 (0.98 to 1.01)	1.00 (0.98 to 1.01)	1.01 (0.99 to 1.02)	1.01 (0.99 to 1.02)	1.00 (0.98 to 1.01)
T2 lesions volume change^a^ at 96 weeks	1.00 (0.99 to 1.01)	1.01 (0.99 to 1.02)	1.00 (0.99 to 1.01)	1.00 (0.99 to 1.01)	1.01 (0.99 to 1.02)	1.00 (0.98 to 1.01)	1.01 (0.99 to 1.02)	1.00 (0.98 to 1.01)
cCEL at 48 weeks^a^	1.01 (0.99 to 1.05)	1.02 (0.99 to 1.04)	1.01 (0.99 to 1.04)	1.01 (0.99 to 1.04)	1.02 (0.99 to 1.05)	1.01 (0.96 to 1.05)	1.03 (0.99 to 1.07)	0.99 (0.93 to 1.04)
cCEL at 96 weeks^a^	1.01 (0.99 to 1.03)	1.01 (0.99 to 1.03)	1.01 (0.99 to 1.02)	1.01 (0.99 to 1.02)	1.02 (0.99 to 1.04)	1.02 (0.99 to 1.06)	1.02 (0.99 to 1.06)	0.99 (0.93 to 1.03)
cNT2 at 48 weeks^a^	1.01 (0.99 to 1.03)	1.02 (0.99 to 1.06)	1.01 (0.98 to 1.04)	1.02 (0.99 to 1.05)	1.03 (0.99 to 1.06)	1.03 (0.99 to 1.06)	1.01 (0.98 to 1.04)	0.99 (0.95 to 1.03)
cNT2 at 96 weeks^a^	1.01 (0.99 to 1.03)	1.02 (0.99 to 1.04)	1.00 (0.98 to 1.02)	1.01 (0.99 to 1.03)	1.02 (0.99 to 1.04)	1.02 (0.99 to 1.05)	1.01 (0.99 to 1.04)	0.99 (0.96 to 1.02)

MSIS-29: Multiple Sclerosis Impact Scale; SF-36: 36-Item Short Form Health Survey; PCS: Physical Component Summary; MCS: Mental Component Summary; EDSS: Expanded Disability Status Scale; 3M: 3 months; CDP: confirmed disability progression; T25FW: Timed 25 Foot Walk; NHPT: Nine Hole Peg Test; SDMT: Symbol Digit Modalities Test; NBV: normalized brain volume; NCGMV: normalized cortical gray matter volume; NWGMV: normalized whole gray matter volume; cCEL: cumulative number of contrast enhancing lesions; cNT2: cumulative number of new or newly enlarging T2 lesions.

The table shows odds ratios with their 95% confidence intervals (in brackets), significant associations are shown in bold type.

a Per unit increase.

Table 6 shows a summary of four selected logistic regression models with significant associations between HRQOL outcomes and significant disability worsening. Significant worsening on the physical outcome measures EDSS, T25FW, and NHPT was strongly associated with worsening on physical HRQOL. Significant worsening on the SDMT at 48 weeks was strongly associated with worsening on the MSIS-29 Psychological score (odds ratio = 6.91, 95% confidence interval = 2.00 to 27.47), although this estimate is less precise and has a wide confidence interval, likely because there were only few SDMT 3M CDP worsening events (Tables 2 and 6).

**Table 6.** Detailed results from four selected logistic regression models.

Predictor variables	Odds ratio	95% confidence interval	*p*
EDSS 3M CDP at 48 weeks and SF-36 MCS worsening at 48 weeks			
SF-36 PCS at baseline^a^	1.07	1.05 to 1.10	< 0.0001
Male sex (reference: female)	1.35	0.90 to 2.00	0.15
Age [years]^a^	0.98	0.96 to 1.01	0.18
Trial arm: natalizumab (reference: placebo)	0.96	0.65 to 1.40	0.82
EDSS at baseline^a^	1.05	0.86 to 1.28	0.64
EDSS 3M CDP at 48 weeks:			
No (reference)	1.00	(reference)	–
Yes	1.99	1.13 to 3.43	0.01
T25FW 3M CDP at 48 weeks and MSIS-29 Physical worsening at 48 weeks			
MSIS-29 Physical at baseline^a^	0.97	0.96 to 0.97	< 0.0001
Male sex (reference: female)	1.03	0.70 to 1.53	0.86
Age [years]^a^	1.02	0.99 to 1.05	0.15
Trial arm: natalizumab (reference: placebo)	1.26	0.87 to 1.82	0.23
T25FW at baseline [s]^a^	1.04	1.01 to 1.07	0.003
T25FW 3M CDP at 48 weeks:			
No (reference)	1.00	(reference)	–
Yes	3.55	2.36 to 5.36	< 0.0001
NHPT 3M CDP at 48 weeks and MSIS-29 Physical worsening at 48 weeks			
MSIS-29 Physical at baseline^a^	0.97	0.96 to 0.98	< 0.0001
Male sex (reference: female)	0.95	0.65 to 1.38	0.77
Age [years]^a^	1.02	0.99 to 1.04	0.20
Trial arm: natalizumab (reference: placebo)	1.23	0.86 to 1.75	0.27
NHPT at baseline [s]^a^	1.01	0.99 to 1.02	0.19
NHPT 3M CDP at 48 weeks:			
No (reference)	1.00	(reference)	–
Yes	2.99	1.38 to 6.55	0.005
SDMT 3M CDP at 48 weeks and MSIS-29 Psychological worsening at 48 weeks			
MSIS-29 Psychological at baseline^a^	0.98	0.97 to 0.99	< 0.0001
Male sex (reference: female)	1.43	0.94 to 2.16	0.09
Age [years]^a^	1.02	0.99 to 1.05	0.22
Trial arm: natalizumab (reference: placebo)	1.02	0.68 to 1.53	0.92
SDMT at baseline^a^	1.00	0.99 to 1.02	0.94
SDMT worse at 48 weeks:			
No (reference)	1.00	(reference)	–
Yes	6.91	2.00 to 27.47	0.003

EDSS: Expanded Disability Status Scale; 3M: 3 months; CDP: confirmed disability progression; SF-36: 36-Item Short Form Health Survey; MCS: Mental Component Summary; PCS: Physical Component Summary; T25FW: Timed 25 Foot Walk; MSIS-29: Multiple Sclerosis Impact Scale; NHPT: Nine Hole Peg Test; SDMT: Symbol Digit Modalities Test.

a Per unit increase.

## Discussion

Selecting the most informative primary outcome measure is an important part of designing the best possible clinical trial in SPMS. Currently, the EDSS is the standard disability outcome measure in all forms of MS. We previously compared the EDSS to the newer outcome measures T25FW and NHPT and showed that the T25FW may be the more useful primary outcome measure for clinical trials in SPMS, because it records more worsening events per unit of time, which has the potential to reduce the duration and therefore the cost of clinical trials.^23^ In an additional investigation comparing worsening events to similarly defined improvement, we also showed that the T25FW may be more reliable and less prone to random variations and measurement errors than the EDSS.^24^ This current investigation on the HRQOL impact and patient-relevance of a variety of clinical and imaging outcome measures showed that worsening on the EDSS and T25FW is most consistently related to worsening HRQOL, both with regard to physical and psychological HRQOL and for both MSIS-29 and SF-36.

Our investigation on the association between HRQOL measures and clinical and imaging outcome measures showed that the EDSS and T25FW were associated with most HRQOL measures used here, while the NHPT and SDMT were less consistently associated, and the investigated lesional and volumetric MRI outcomes were not at all associated with worsening HRQOL.

These findings are in agreement with a smaller longitudinal study on 132 people with SPMS or primary progressive MS that investigated the association of MSIS-29 Physical worsening with worsening on disability outcomes T25FW, EDSS, and NHPT. In that study, only the T25FW, but not the EDSS or NHPT, was significantly associated with MSIS-29 Physical worsening after a mean follow-up of 5 years.^25^ Another smaller longitudinal study including 57 people with SPMS similarly showed the T25FW, but not the EDSS or NHPT, to be associated with MSIS-29 Physical and MSIS-29 Psychological worsening at 2 years of follow-up.^26^ These findings support the conclusion that the T25FW is a patient-relevant outcome in SPMS over a follow-up period of several years.

Our investigation of lesional and volumetric MRI outcomes showed no significant relationships with HRQOL worsening. There is relatively fewer studies on the topic of the impact of MRI changes on HRQOL. The previously mentioned smaller longitudinal study including 57 people with SPMS showed that lesional and volumetric MRI measures were not related to MSIS-29 subscores, whereas some magnetization transfer ratio (MTR) measures were significantly associated with the worsening MSIS-29 Psychological subscore at 2 years of follow-up.^26^ Our current analysis does not include MTR measures, so we cannot comment on their HRQOL impact.

This study has several limitations. While ASCEND is a large trial dataset with almost 900 participants, the included individuals fulfill the specific inclusion criteria of the original trial, and it is uncertain whether our conclusions from this pre-selected cohort can be generalized to the general populations of people with SPMS. ASCEND also had a relatively large number of participants, of which 26% of the cohort dropped out of the trial by the end of follow-up,^15^ which may have affected the precision of our analyses. Although the dataset contained information on several modern MRI outcomes, we cannot comment on the impact of regional lesional or volumetric measures in the brainstem or spinal cord, which were not included in our data source. Similarly, we cannot comment on the effect of changes in symptomatic medications during this study. Our analyses should be confirmed in other clinical trial datasets and real-world clinical cohorts.

Finding new and effective treatments for SPMS remains a significant and largely unmet challenge. Many more clinical trials will likely be necessary to develop such treatments, and their design should involve the most useful primary outcome measure. We previously showed that the T25FW to be more sensitive^23^ and more reliable^24^ than the established EDSS. Another study in a large dataset from the placebo arms of clinical trials showed that T25FW worsening is a good predictor of EDSS worsening, which argues for the usefulness of the T25FW to shorten the duration of clinical trials.^27^ Our current investigation adds to this that the T25FW and EDSS have a similar impact on two widely used HRQOL measures, and therefore similar patient-relevance in a 2-year clinical trial. The association of disability measures, MRI outcomes, and HRQOL measures should be investigated in other trial datasets and clinical cohorts in progressive and relapsing-remitting MS.
